# Flexible Orientation Tuning of Visual Representations of Human Body Postures: Evidence From Long-Term Priming

**DOI:** 10.3389/fpsyg.2020.00393

**Published:** 2020-03-10

**Authors:** Karl Verfaillie, Anja Daems

**Affiliations:** Laboratory of Experimental Psychology, Brain and Cognition, Faculty of Psychology and Educational Sciences, KU Leuven, Leuven, Belgium

**Keywords:** visual perception of body postures, long-term priming, orientation dependence, actor identity, prototypical

## Abstract

The proficiency of human observers to identify body postures is examined in three experiments. We use a posture decision task in which participants are primed with either anatomically possible or impossible postures (in the latter case the upper and lower body face in opposite directions). In a long-term priming paradigm (i.e., in an initial priming block of trials and a subsequent test phase several minutes later), we manipulate the relation between priming and test postures with respect to the identity of the person in the body postures (Experiment 1), the prototypicality of the depth orientations (Experiment 2), and the variability of the priming orientations (Experiment 3). Reaction time to the test postures is the main dependent variable. In Experiment 1 it is found that priming of postures does not depend on the exact visual appearance of the actor (either same priming and test female or male figure or different figures), supporting the hypothesis that posture priming primarily is determined by the spatial relations between the body parts and much less by characteristics of the person involved. Long-term priming in our paradigm apparently is based on the reactivation of high-level posture representations that make abstraction of the identity of the human figure. In Experiment 2 we observe that privileged or prototypical orientations (e.g., 3/4 views) do not affect long-term priming of body postures. In Experiment 3, we find that increasing or decreasing the variability between the priming and test figures influences reaction time performance. Collectively, these results provide a better understanding of the flexibility (e.g., invariant to identity) and limits (e.g., depending on depth orientation) of the processes supporting human posture recognition.

## Introduction

**FIGURE 1:**
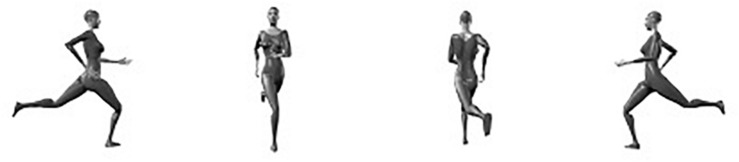
A snapshot of a running action seen from 4 different viewpoints.

Human observers exhibit an impressive level of proficiency in identifying the body postures of conspecifics (e.g., Daems and Verfaillie, 1999; Rumiati, 2000; Willems et al., 2014 (much like the recognition of faces; e.g., Galton, 1883; Maurer et al., 2002; Van Belle et al., 2010a, b; Verfaillie et al., 2014; Vrancken et al., 2019, although the issue whether face and body recognition are “special” is under debate, e.g., Gauthier et al., 1999; Tai et al., 2004; Reed et al., 2012). On the one hand, this is important from an evolutionary point of view, because posture identification frequently is crucial for adequately interpreting the intention of the interacting partner, which in itself is important for reacting in a socially appropriate manner (e.g., Jellema and Perrett, 2003; Gallese et al., 2004; Sebanz and Frith, 2004; Blake and Shiffrar, 2007; Brooks et al., 2008; Manera et al., 2010, 2011; Brown and Brüne, 2012; Moors et al., 2015; Isik et al., 2017; Vrancken et al., 2017; Wang et al., 2018). On the other hand, the identification of other people’s postures is not trivial from a perceptual standpoint (e.g., Gold et al., 2008). Depending on the relation between the acting body and the observer, the same body posture can result in a multitude of possible visual projections (e.g., Verfaillie and Daems, 2002; Chan et al., 2004; de la Rosa et al., 2013; Ballarini and Thornton, 2017). For instance, human observers automatically and effortlessly identify the body postures shown in Figure 1 as snapshots of a female person running, even though the proximal stimuli are radically different.

**FIGURE 2:**
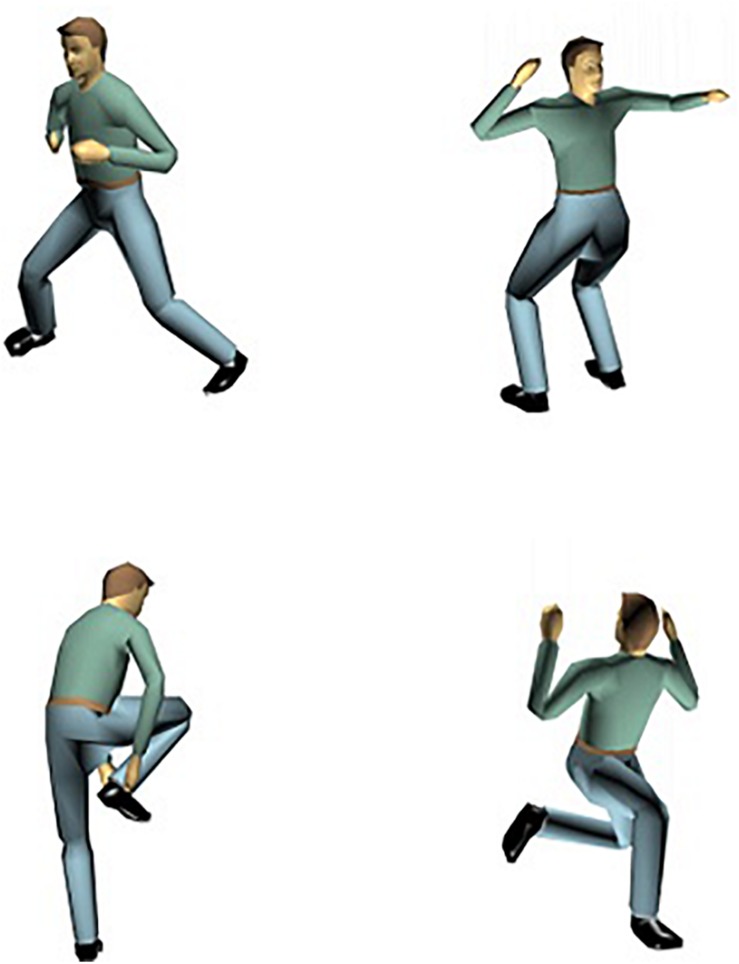
Examples of anatomically possible (first column) and impossible (second column) postures.

**FIGURE 3:**
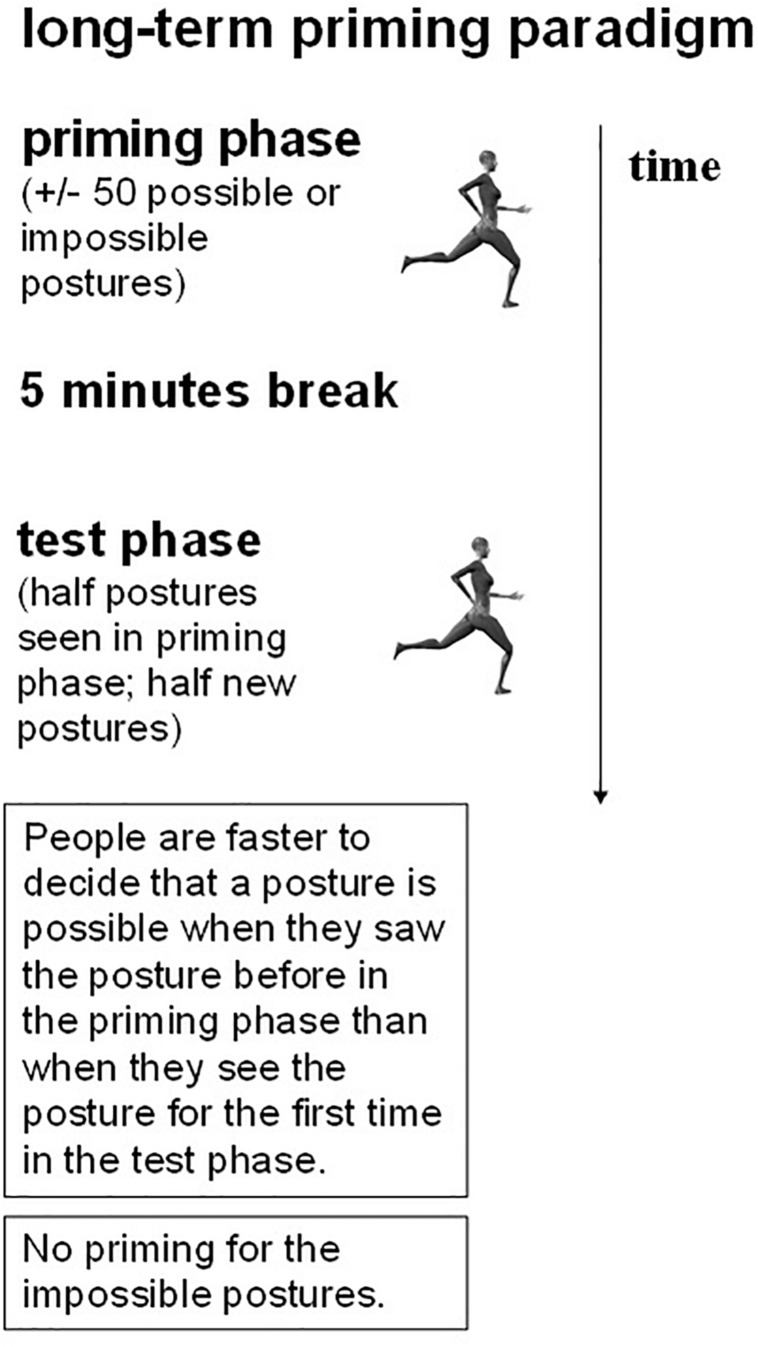
Illustration of the basics of the long-term priming paradigm.

In order to investigate the nature of the representations underlying visual perception of human body postures, Daems and Verfaillie (1999), Experiments 3 and 4) developed a posture decision task in combination with a long-term priming procedure. On each trial, in an initial priming block, a static picture of a particular human body posture was shown. In half of the pictures, an anatomically possible pose was presented; in the other half of the trials an anatomically impossible (i.e., the upper-waist body part of the actor was rotated 180° around the top-bottom axis, so that upper and lower body parts were facing in opposite directions; see Figure 2 for examples) was shown. Participants had to decide whether a posture was anatomically possible or not and reaction time (RT) was registered. After a 5 min break, subjects saw a second, testing, block of anatomically possible and impossible postures (Figure 3; we provide more details on the procedure in future sections). Some of the test postures were already presented in the priming block, whereas other postures were new. Daems and Verfaillie (1999) observed a long-term priming effect: In the testing phase, participants were on average about 35 ms faster to decide that a posture was anatomically possible when they had seen the posture before in the priming block than when they encountered the posture for the first time in the testing block.

Daems and Verfaillie (1999; Experiment 4) examined how sharply tuned the representations of a body posture are to a particular orientation in depth. To this end, the depth orientation difference between priming and test posture was varied parametrically. All participants saw exactly the same test postures, but, for a given test posture, a specific participant either saw no related posture in the priming phase or priming and primed postures that differed by a 0, 15, 30, 45, or 60° depth rotation around the body’s top-bottom axis. For anatomically possible postures, the facilitatory priming effect of about 30 ms was replicated in the same-prime condition (0° difference). In the condition with a depth rotation of only 15°, the priming effect decreased to a (non-significant) 15 ms. After a depth rotation of only 30° or more, the priming effect disappeared completely. This finding suggests that visual representations of human postures are viewpoint-dependent and finely tuned to a particular depth orientation. One of the purposes of the present study is to further examine this depth orientation tuning of posture representations.

Our underlying working hypothesis is that priming results from the persistent activation of representations that mediate perceptual organization of the posture. In order to perform the posture decision task during the priming phase, participants compute a representation of the posture and, when they re-encounter the posture during the test phase, activation of the representation is facilitated, resulting in shorter reaction times. The finding that even a relatively moderate depth rotation of the body posture between priming and test phase already results in a drastic reduction of facilitatory priming suggests that the underlying representations that mediate visual posture identification are sharply tuned to specific depth orientations.

However, there is an alternative explanation. Facilitatory priming was strongly dependent on the repetition of exactly the same priming posture in all its details. Therefore, it is possible that priming was based on early, lower-level stimulus-specific representations that are only precursors to higher-level body representations. The observation that there was no facilitatory priming for impossible postures (see Nilsson et al., 1992; Peigneux et al., submitted), not even in the case of an identical prime-view, runs counter to this objection. Moreover, there is evidence (e.g., Cave et al., 1996; see Raffone et al., 2014, for more general related issues) that long-term priming reflects the characteristics of high-level representations, rather than lower levels of representation (although this is under debate, e.g., Srinivas, 1995) footnote 1. In Experiment 1, we tested this alternative hypothesis more directly by manipulating the visual appearance of the actor performing the posture. In Experiment 2, we examined whether privileged posture orientations (i.e., 3/4 views) could explain the divergent results between Experiment 4 of Daems and Verfaillie (1999) and the present Experiment 1. In Experiment 3, we investigated whether increasing or decreasing the variability in orientation differences between the priming figures influences subsequent priming in the test phase. The theoretical rationale relates to the potential importance of similarity of priming stimuli in the priming phase for flexible identity and orientation tuning of the postures.

## Experiment 1

Long-term priming was examined with the paradigm developed by Daems and Verfaillie (1999) (see Figure 3). Participants performed a posture decision task in a priming block of trials, followed by a test phase. All participants saw the same test postures and the RT to decide whether a test posture was anatomically possible or not was the dependent variable. The relation between priming and test postures was manipulated in two ways. Only the possible postures were systematically involved in these manipulations; the impossible postures served as filler stimuli.

**FIGURE 4:**
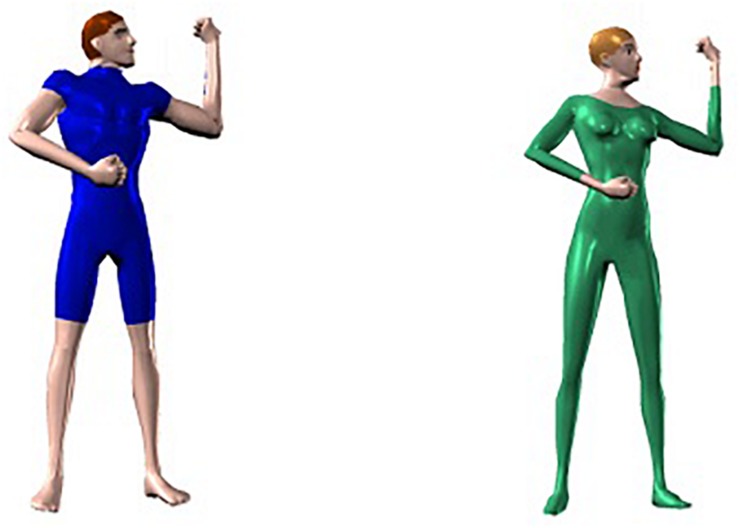
The two actors (one male, one female) shown in Experiment 1.

**FIGURE 5:**
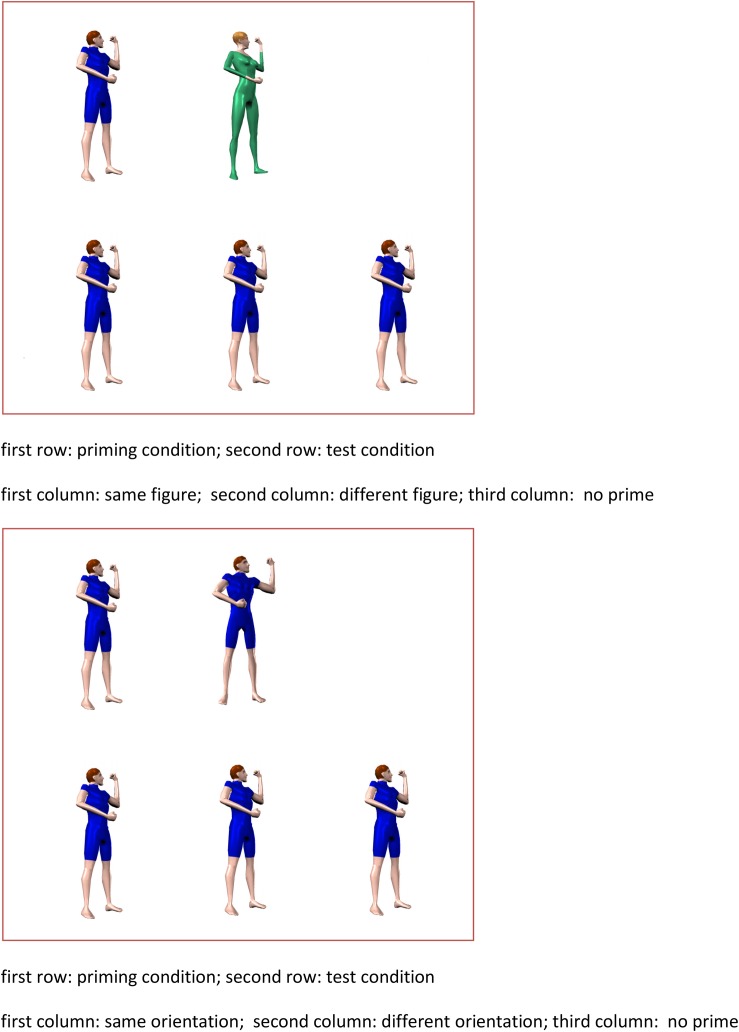
Illustration of the priming conditions in Experiment 1. Top panel: same orientation/different figure condition; bottom panel: different orientation/same figure condition. First row in first panel: priming condition (same or different figure and same orientation, or no prime). First row in second panel: priming condition (same figure and same or different orientation, or no prime). Second row in both panels: primed condition (here figure in 45° orientation).

First, and most importantly for the present experiment, we manipulated the visual appearance of the actor involved in the postures. As shown in Figure 4, the human model either had a typical male build, had short brown hair, was wearing a dark blue trouser suit with short sleeves and trouser-legs, and was bare-footed, or had a typical female build, had medium blond hair, and was wearing a light green trouser suit with long sleeves and legs and green shoes. As shown in Figure 5, a posture in the test phase was personated by the same male or female actor in the priming block (same-figure prime), was personated by the other figure (different-figure prime), or was not shown during the priming phase (baseline no-prime condition). On the one hand, a body posture is determined primarily by the spatial relations between the body parts and much less by characteristics of the person performing the posture, such as her or his gender, body proportions, clothing, or hair color. If long-term priming in our paradigm is based on the reactivation of high-level posture representations that make abstraction of the identity of the human figure, priming (i.e., faster RT in the priming condition than in the baseline no-prime condition) should be observed both with same-figure primes and with different-figure primes. On the other hand, the image of the female model in a particular posture differs drastically from the image of the male model in the same posture (or vice versa). If long-term priming can be traced back to the activation of early, low-level representations, priming should be absent or at least substantially reduced when the model changes from priming to test phase.

The second purpose of Experiment 1 was an attempt to replicate the strong viewpoint dependence observed by Daems and Verfaillie (1999). The trunk of the actor in the test phase always was oriented 45° or 225° to the right. In the priming block of trials, a posture was either shown in the same depth orientation or in an orientation that differed by a depth rotation of 30° around the actor’s top-bottom axis, resulting in an orientation of 15 or 195°. As shown in the top row of the bottom panel of Figure 5, the difference between postures in a 45 and a 15° orientation (which also holds for the difference between a 225 and a 195° orientation) was quite subtle. Nevertheless, in an experiment with a similar manipulation, Daems and Verfaillie (1999) observed that a depth rotation of only 30° between priming and test posture was sufficient to reduce the priming effect to a non-significant 6 ms benefit in comparison to a no-prime baseline. We therefore predicted reliable priming in the same-view condition and no priming in the 30°-difference condition.

For a given posture there were four different conditions: same figure/same orientation in priming and test block, different figure/same orientation, same figure/different orientation, and no prime. In principle, both the visual appearance of the human model and the precise depth viewpoint from which a posture is observed are irrelevant to perform the posture decision task. The observation that long-term priming with same figure/same orientation primes generalizes over changes in characteristics of the model but not over changes in the depth orientation of the posture would support the hypothesis that the high-level representations of human postures are viewpoint specific. Moreover, the observation that same-orientation prime-test pairs produce (relatively) equivalent facilitatory effects independently of the precise visual appearance of the actor (either same priming and test female or male figure or different figures) would indicate that posture priming primarily is determined by the spatial relations between the body parts and much less by characteristics of the person involved in the posture. This would imply that high-level posture representations to a large extent make abstraction of the identity of the human figure.

### Method

#### Participants

Sixty-four first-year students (who received course credit) or other undergraduate and graduate students (who were paid) participated in the experiment. They were tested individually. The subjects gave informed consent in accordance with the declaration of Helsinki. All participants had normal or corrected-to-normal vision and were naive with respect to the hypothesis under investigation.

#### Stimuli and Apparatus

The experimental stimulus set consisted of eight sets of 24 static full-color pictures of anatomically possible human body postures. In the first set, a male bare-footed model with short brown hair and a blue trouser suit with short sleeves and trouser-legs was depicted in 24 different postures with the trunk in a 15° orientation in depth (with 0° corresponding to a frontal view). In the second set, the same model was shown in the same 24 postures, but now in a 45° depth orientation. The third and the fourth set portrayed the human figure in 24 new postures, with the trunk in a 195 and a 225° depth orientation, respectively. In the four remaining sets, the same postures were shown as in the previous four sets, but the model now was a female person with medium blond hair wearing green shoes and a green trouser suit with long sleeves and legs. Care was taken that as many body parts as possible were visible in all views.

In addition to the experimental stimulus set, 60 filler stimuli of 40 anatomically impossible postures were constructed. In these impossible poses, the above-waist part of the body was rotated 180° in depth around the model’s top-bottom axis, so that the figure’s above-waist part was oriented in exactly the opposite direction vis-a-vis the below-waist part of the body (Figure 2). The impossible poses were built from poses that were different from the possible postures in the experimental stimulus set. Half of the impossible stimuli showed the male model and the other half showed the female model. The trunk was in a 15° or a 195° depth orientation or in a 195 or 225° depth orientation.

The training stimuli consisted of the 14 training stimuli used in Experiments 3 and 4 of Daems and Verfaillie (1999).

All anatomically possible and impossible postures were created using the Poser software package (Fractal Design Corporation, 1996) and then improved with graphics software. During the experiment, stimuli were presented with a computer equipped with a VGA graphics card on a 15-inch computer screen. Stimuli were viewed binocularly at a comfortable viewing distance of approximately 65 cm. In a standing up pose, the human figure subtended approximately 10.5° of visual angle. A response box with two buttons with breaking contacts was connected to the PC.

#### Procedure and Design

Each trial started with the presentation of an auditory warning signal and a fixation cross in the middle of the screen. After 500 ms, the stimulus appeared and participants had to decide as rapidly as possible whether the depicted body posture was anatomically possible or not, by pressing one of two response keys. Half of the participants pressed the right button for possible poses and the left button for impossible poses, while this stimulus-response mapping was reversed for the other half of the subjects. Auditory feedback was given by means of a high-pitch tone for a correct answer and a low-pitch tone for an incorrect answer. The picture was presented until participants responded, except when the RT exceeded 2 s, in which case the trial was ended.

Participants performed this posture decision task with the 14 training stimuli, immediately followed by the priming block of 68 priming stimuli (36 experimental, anatomically possible postures and 32 filler, impossible postures) presented in an individually determined random order. After a 5 min break (during which the experimenter and the subject had an informal conversation), the testing block of 88 stimuli (48 experimental, anatomically possible postures and 40 filler, impossible postures) was administered, again in a random presentation order. During instructions, it was never mentioned that subjects were involved in a priming experiment.

There were four conditions (Figure 5), determined by the relation between priming and test postures: a same figure/same orientation condition, a different figure/same orientation condition, a same figure/different orientation condition, and a no-prime condition. The eight experimental stimulus sets were divided in eight groups of three stimuli that were rotated across conditions and participants, in such a way that each stimulus appeared equally often in each of the four conditions. Each participant saw 12 postures in each condition. In the same figure/same orientation condition, half of the postures were personated by the female model and half were personated by the male model, both in the priming phase and in the test phase. In the different figure/same orientation condition, half of the postures were personated by the female model in the priming phase and by the male model in the test phase, whereas the other half of the postures were personated by the male model in the priming phase and by the female model in the test phase (first two columns in the top panel of Figure 5 for examples of same/different figures in the same orientation). In these two conditions, the postures were always shown in the same depth orientation (45 or 225°) in priming and test block. In the same figure/different orientation condition, half of the postures were personated by the female model and half by the male model, and for a given posture the model was constant over the priming and the test block (first two columns in the bottom panel of Figure 5 for examples of same figures in different orientations). The depth orientation of the priming posture differed by 30° from the test posture. Finally, in the no-prime condition, half of the postures were personated by the female model and half by the male model and were shown for the first time in the test block (third column in both panels of Figure 5).

All participants saw the same anatomically impossible filler stimuli. During the priming block and the test block, 32 and 40 impossible postures were shown, respectively, half of them personated by the male model and half by the female model. In both phases, half of the male and half of the female postures were oriented (more or less) toward the viewer (15 or 45° in the priming phase and 45° in the test phase) and half were oriented (more or less) away from the viewer (195 or 225° in the priming phase and 225° in the test phase). Twelve impossible postures were shown by the same figure and in the same depth orientation in priming and test phase, 10 impossible postures were performed by the same figure but shown from a different viewpoint in the test phase, and 10 impossible postures were shown from the same viewpoint, but were performed by the other person in the test phase. The remaining eight impossible filler stimuli were only administered during the test phase.

### Results

The dependent variable was the RT to the (anatomically possible) test postures. Trials in which the stimulus was not identified correctly either in the priming or in the test block and trials in which the RT fell below a cut-off value of 200 ms or above a cut-off value of 2000 ms were discarded from the RT data set (approximately 1% of the data set). The remaining RTs were entered in a subject repeated-measures analysis of variance (ANOVA) with priming condition (identical prime, different figure but same orientation, same figure but different orientation, and no prime) as a within-subject variable and participant group as a between-subjects variable, and in a stimulus ANOVA with priming condition as within-stimulus variable and stimulus group as between-stimuli variable. (Especially in psycholinguistic research, but also in perception research, it is informative to perform both subject and stimulus analyses and present them together, Kirk, 1968; Pittenger, 2003, as we did in previous analyses of experiments with the same paradigm; Daems and Verfaillie, 1999; Verfaillie and Daems, 2002.) The mean RTs are shown in Table 1. The MS errors in the ANOVAs give an indication of the variability.

**TABLE 1:** Mean identification time (in ms) of anatomically possible human postures in the test phase of Experiment 1 as a function of long-term priming condition.

Long-term priming condition	RT to possible body postures
Same figure/same orientation	615
Different figure/same orientation	624
Same figure/30° different orientation	621
No priming	646

Both the subject and the stimulus analysis revealed a statistically significant main effect of priming condition (4 levels: same figure, same orientation; different figure, same orientation; same figure, different orientation, no prime), *F*_1_(3,168) = 6.04, *MSe* = 1989, *p* < 0.01, and *F*_2_(3,120) = 6.24, *MSe* = 1890, *p* < 0.01. Dunn’s multiple comparison test showed that RTs in the no-prime baseline condition were significantly longer than in the identical same-figure/same-orientation condition, *tD*_1_ = 3.64, *MSe* = 2339, *p* < 0.01, and *tD*_2_ = 3.97, *MSe* = 1812, *p* < 0.01, in the different-figure/same-orientation condition, *tD*_1_ = 2.74, *MSe* = 2229, *p* < 0.05, and *tD*_2_ = 2.83, *MSe* = 2441, *p* < 0.05, and in the same-figure/different-orientation condition, *tD*_1_ = 3.52, *MSe* = 1686, *p* < 0.01, and *tD*_2_ = 3.29, *MSe* = 1949, *p* < 0.01. The differences between RTs in the identical condition and the different-figure condition and between RTs in the identical condition and the different-orientation condition were not significant.

Note that the long-term priming effects in the test phase were not caused by (accidental) differences in exposure times to the initial, priming postures. Indeed, a subject and stimulus analysis on the RTs in the priming phase showed that long-term priming condition had no effect, *F*_1_(2,112) = 0.49, *MSe* = 3222, *p* > 0.60, and *F*_2_(2,80) = 1.22, *MSe* = 4273, *p* < 0.30. Mean RTs to postures that later appeared in the identical condition, the different figure condition, and the different orientation condition were 873, 882, and 875 ms, respectively.

### Discussion

First, in comparison to the no-prime baseline, participants were faster to decide that a posture was anatomically possible when they had seen that posture several minutes earlier during the priming phase. Most importantly, this priming effect was not significantly larger when the human model in the priming posture was identical to the model in the test phase than when priming and test postures were personated by distinctly different human models. Apparently, facilitatory long-term priming in the posture decision task is not contingent upon the repetition of exactly the same stimulus person. This supports the hypothesis that long-term priming is based on the re-activation of high-level representations of human body postures (rather than being based on an early, low-level representation of the stimulus) that make abstraction of the precise visual appearance of the human figure.

Second, contrary to our expectations, priming also generalized over an orientation difference of 30° between priming and test posture. On the one hand, given the fact that the difference between postures in a 45 and a 15° orientation and between a 225 and 195° orientation was quite subtle (see Figure 5 for examples), this is not surprising. On the other hand, in a similar experiment, Daems and Verfaillie (1999) did not observe significant facilitatory priming with a depth orientation difference of 30° between priming and test posture. For comparison, in Experiment 4 of Daems and Verfaillie (1999), the priming effect (difference with the no-prime baseline) amounted to 27 ms in the identical condition (31 ms in the present study), but only to 6 ms in the condition with a 30° orientation difference (25 ms in the present study). The experiment of Daems and Verfaillie (1999) suggests that posture representations are very sharply tuned to a particular orientation in depth, while the present study suggests that the orientation tuning of action representations is broader (or at least dependent on stimulus or task conditions). One of the purposes of Experiments 2 and 3 was to test possible accounts for these divergent findings.

## Experiment 2

A possible explanation for the contradictory findings in our previous experiments hinges on the hypothesis that some views of body postures might have a privileged status over other views and that posture recognition proceeds through the activation of these privileged or prototypical orientations. Evidence mainly comes from studies of object recognition.

First, there is ample evidence that some views of three-dimensional, familiar objects are rated as more canonical or prototypical than other views and that objects depicted in canonical orientations are identified more easily (e.g., faster) than when shown from less canonical angles (e.g., Palmer et al., 1981; Verfaillie and Boutsen, 1995; Lawson and Humphreys, 1996, 1998; Boutsen et al., 1998; Blanz et al., 1999; Ghose and Liu, 2013; Alshehri et al., 2018; but see Cutzu and Edelman, 1994, and Niemann et al., 1996, who did not find evidence for universally valid canonical views for novel objects). Stable views (e.g., a 3/4 view) typically are views in which small changes in depth orientation do not lead to prominent changes in the projected image of the object and that are most informative about the identity of an object (e.g., because the most diagnostic object parts are clearly visible; see Verfaillie and Boutsen, 1995, for a more detailed discussion). It is possible that body postures (and not only objects in general) in a 3/4 depth orientation also have a privileged status.

Second, it has been suggested that identification of objects, even objects viewed from unconventional viewing angles, is achieved by activating (a number of) neighboring prototypical views (for an overview on this discussion, see Bülthoff et al., 1995; Ghose and Liu, 2013). For instance, using a long-term priming paradigm, Srinivas (1993) reported that having seen an object shown in an unusual orientation during the priming phase produced almost as much facilitation to identify that object shown in a usual orientation in the test phase as having seen the same object in the same (usual) orientation during the priming phase. Having seen an object in a usual orientation during the priming phase, in contrast, did not facilitate later recognition of the object in an unusual orientation in the test phase. Apparently, processing an object seen from an unusual viewpoint in the priming phase involved the activation of a representation of the object in a neighboring, more prototypical view, resulting in facilitatory priming during the test phase (see Perrett et al., 1989; Perrett et al., 1991, for related neurophysiological findings).

The test postures in Experiment 4 of Daems and Verfaillie (1999) were always in a less prototypical orientation (75 or 255°), whereas the test postures in Experiment 1 of the present study were in a more prototypical orientation (45 or 225°). This might explain why, when priming and test posture differed by a 30° depth rotation, facilitatory priming was observed in the latter experiment but not in the former experiment. If the discrimination between possible and impossible body postures in less prototypical orientations indeed involves the activation of neighboring prototypical orientations, the less prototypical 15 and 195° priming postures (the priming postures in the 30° different conditions in Experiment 1 of the current study) would result in priming during the test phase, whereas the prototypical 45 and 225° priming postures (the priming postures in the 30° different conditions in Daems and Verfaillie’s Experiment 4) would not.

In Experiment 2, the test postures were shown either in a prototypical 45 or 225° orientation (further referred to as the three-quarter views) or in a less prototypical 75 or 255° orientation (further referred to as the sagittal views, even though strictly spoken the views only approximate the 90 and 270° sagittal views; note that one of the reasons for choosing these views close to the sagittal views instead of the exact sagittal views is that body parts that were occluded in the sagittal views mostly became visible in the close-to-sagittal views). These test postures were preceded by a posture in the same view, the same posture in a view that differed by a rotation of 30° (15 or 195° and 45 or 225°), or were not shown during the priming phase. Of crucial importance is the condition in which priming and test postures differed by 30°. As spelled out in previous paragraphs, facilitatory priming (i.e., shorter RTs in comparison to the no-prime condition and RTs at the same level as in the identical prime condition) was predicted in this condition for test postures in prototypical three-quarter orientations but not for test postures in less prototypical sagittal orientations. The underlying rationale is that the 30° different prime preceding the three-quarter test posture is a less prototypical view and processing this view also activates the three-quarter view, whereas the 30° different prime preceding the sagittal test posture is a prototypical view and processing this view does not lead to activation of less prototypical views. Support for this hypothesis would imply that, first, postures in some orientations have a more privileged status than postures in other orientations and, second, posture decision proceeds through the activation of neighboring privileged views.

### Method

#### Participants

A total of 84 first-year students psychology at the Leuven University with normal or corrected-to-normal vision participated for course credit. Participants were tested individually.

#### Stimuli

**FIGURE 6:**
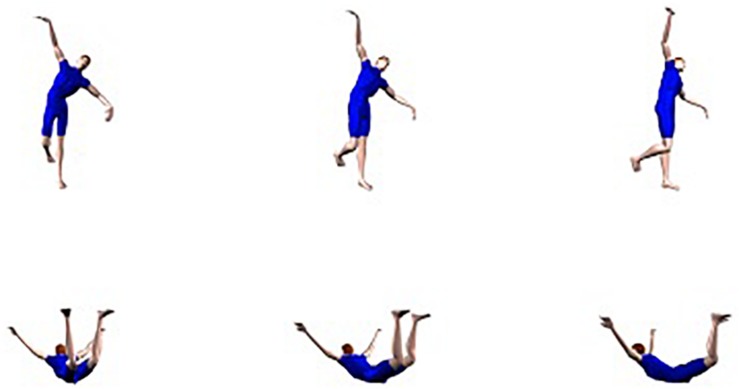
Example of the stimuli used in Experiment 2. The trunk is in a 15°, 45°, or 75° degrees depth orientation (top row) or a 195°, 225°, or 275° depth orientation (bottom row).

The experimental stimulus set consisted of six sets of 24 color pictures of a male figure. The first three sets depicted the figure in 24 different anatomically possible body postures with the trunk in a 15, 45, or 75° depth orientation, respectively. In the other three sets, the same figure was shown in 24 other postures in a 195, 225, or 275° depth orientation, respectively. Figure 6 depicts an example of two postures, each in three different depth orientations. In addition to the experimental stimuli, 48 filler stimuli of 36 different anatomically impossible postures were created in the same way as in Experiment 1. The lower body part was in a 15, 45, 75, 195, 225, or 255° orientation in depth.

#### Procedure and Design

Participants performed the same posture decision task as in Experiment 1. All participants saw exactly the same test postures, but different subjects saw different priming postures.

The six sets of experimental stimuli were divided in six groups of four stimuli that were rotated across conditions and across participants. Each participant was presented with eight different postures in each of six conditions. In three conditions, three-quarter views (45 and 225°) were shown during the test phase and in three other conditions, views close to the sagittal orientation (75 and 255°) were shown during the test phase. One third of the three-quarter and quasi sagittal test postures were preceded by the same posture in the same orientation during the priming phase, one third of the test postures were preceded by the same posture rotated by 30° in depth vis-a-vis the test stimulus (i.e., in a 15 and 195° orientation for the three-quarter views and a 45 and 225° orientation for the sagittal views), and one third of the test postures were not shown during the priming phase. All participants saw the same anatomically impossible postures. In half of the impossible postures presented during the test phase, the lower body part half was in a 45 or 255° depth orientation, in the other half, the lower body part was in a 75 or 255° orientation. One third of the impossible test postures were seen in the same depth orientation during the priming phase, one third in a 30°-different orientation, and one third was seen for the first time during the test block of trials. These impossible postures only served as filler stimuli and RTs to impossible postures were not analyzed.

Participants were tested individually. They started the experiment with a block of 14 training stimuli (the same as in Experiment 1), which was followed by a priming block of 56 stimuli presented in a random order (32 possible experimental stimuli and 24 impossible filler stimuli), a 5 min break, and a test block of 84 stimuli in a random order (48 experimental possible stimuli and 36 impossible filler stimuli).

### Results

The RT to decide that a test posture was anatomically possible was the dependent variable. Using the same criteria as in Experiment 1, about 1% of the trials were excluded from analysis. Means of the remaining RTs, as a function of priming condition and test orientation are shown in Table 2 (the MS error values of the ANOVA provide information about the variability).

**TABLE 2:** Mean identification time (in ms) of anatomically possible human postures in the test phase of Experiment 2 as a function of long-term priming condition and test orientation.

	Test orientation	
Long-term priming condition	Three-quarter view	Sagittal view
Same orientation	616	616
30° different orientation	638	625
No priming	658	650

The data were entered in a participant ANOVA with test orientation (three-quarter view or almost sagittal view) and priming condition (same orientation prime, 30° orientation-different prime, or no prime) as within-subject variables and participant group as between-subjects variable, and in a stimulus ANOVA with test orientation and priming condition as within-stimulus variables and stimulus group as between-stimuli variable. The participant and the stimulus analysis yielded a significant main effect of priming condition, *F*_1_(2,156) = 18.68, *MSe* = 3329, *p* < 0.001, and *F*_2_(2,84) = 20.13, *MSe* = 1874, *p* < 0.001. Dunn’s multiple comparison tests showed that, both in the participant and in the stimulus analysis, RTs in the no-prime condition were significantly longer than in the same-orientation condition, *tD*_1_ = 6.40, *MSe* = 6008, *p* < 0.01, and *tD*_2_ = 5.78, *MSe* = 4501, *p* < 0.01, and in the 30°-different condition, *tD*_1_ = 3.64, *MSe* = 6552, *p* < 0.01, and *tD*_2_ = 3.24, *MSe* = 4298, *p* < 0.01. This indicates that there was facilitatory priming, both in the same-orientation condition and in the 30°-different orientation condition. In the stimulus analysis, RTs in the same-orientation condition were shorter than in the 30°-different orientation condition, *tD*_2_ = 3.54, *MSe* = 2447, *p* < 0.01, suggesting that facilitatory priming was more pronounced in the same-orientation condition than in the 30°-different condition. However, this difference was not significant in the participant analysis.

The main effect of test orientation was not significant, *F*_1_(1,78) = 2.25, *MSe* = 2642, *p* > 0.10, and *F*_2_(1,42) = 1.39, *MSe* = 2586, *p* > 0.20, nor was the interaction between test orientation and priming condition, *F*_1_(2,156) = 0.63, *MSe* = 2884, *p* > 0.50, and *F*_2_(2,84) = 1.01, *MSe* = 2083, *p* > 0.30. This is not in line with the predictions. In fact, although not significantly different, RTs to the three-quarter views, which were supposed to have a more privileged status, were slightly longer than RTs to the test postures close to the sagittal view.

Note that again the priming effects in the test phase were not caused by differences in initial identification time during the priming phase, as shown by the absence of main effects of test orientation and priming condition and the absence of an interaction effect in a participant and stimulus analysis on the reaction times in the priming phase. With the three-quarter view test orientation, mean identification time of the priming posture was 862 ms for the same-orientation condition and 852 ms for the 30° different-orientation condition. With the almost frontal view test orientation, mean identification time of the priming posture was 851 ms for the same-orientation condition and 854 ms for the 30° different-orientation condition.

### Discussion

For both test orientations in Experiment 2, we observed long-term priming that was less orientation specific (i.e., priming in the 30° difference condition larger than in the no-prime baseline but smaller than in the identical orientation condition) than in Experiment 4 of Daems and Verfaillie (1999) (where no priming with a 30° orientation difference was found), but more orientation specific than in Experiment 1 of the present study (where priming with a 30° difference, but not different from the identical-orientation condition, was found). Moreover, although not significant, the data in Table 2 suggest that generalization across 30° different orientations was more pronounced with the test postures in an almost sagittal orientation than with the test orientations in a three-quarter view, contrary to what we predicted. It is therefore improbable that the specific test orientations were responsible for the differential orientation tuning effects observed in previous long-term priming experiments.

From a theoretical point of view, these findings have implications for a better understanding of visual representations of human body postures. More specifically, we did not find evidence for the assumption that postures in a three quarter view are processed as prototypical postures nor that body postures in nearby orientations are recognized via the activation of prototypical half-way orientations. Note, however, that the hypothesis that prototypical orientations play a role in the identification of postures in less prototypical orientations can only be rejected with caution. The assumption underlying this hypothesis was that the presence of non-prototypical orientations in the priming phase could result in the activation of similar prototypical views, so that these prototypical orientations are processed more easily later during the test phase. However, this facilitatory effect in principle could also occur when prototypical orientations are shown during the priming phase and non-prototypical orientations during the test phase. In theory it is indeed possible that the presentation of prototypical orientations in the priming phase results in faster activation of these representations in the test phase, facilitating the activation of test postures in non-prototypical orientations that are recognized through the activation of these representations. What does seem to be clear on the basis of Experiment 2, however, is that prototypical orientations are not responsible for the divergent results in Experiment 4 of Daems and Verfaillie (1999) and the present Experiment 1.

## Experiment 3

The design of Experiment 4 in Daems and Verfaillie (1999) and that of Experiment 1 of the present study differ in several respects. In Experiment 3, we tested the effect of one specific difference: the smallest orientation difference between the orientations in the priming phase (in the conditions in which priming and test orientations differed). Indeed, in Experiment 4 of Daems and Verfaillie (1999) the smallest difference between the body postures presented during the priming phase was 15° whereas the smallest difference amounted to 30° in Experiment 1 of the present study. It is possible that smaller differences between different orientations in a stimulus set in the priming phase result in more specific long-term priming effects than larger differences.

**FIGURE 7:**
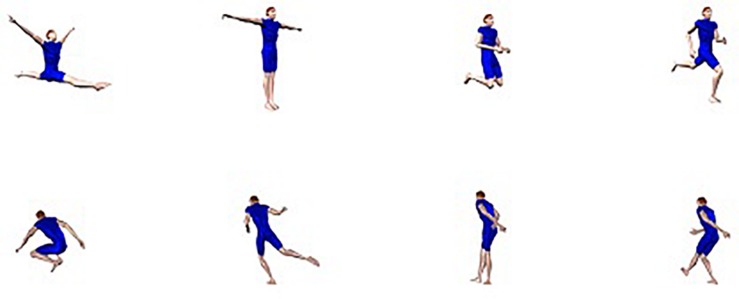
Examples of postures with the trunk in a 45° (first row) or 225° (second row) depth orientation. Note that the orientation of the trunk more or less remains the same in each row.

Under the assumption that different body postures in orientations that are closer to each other are more similar than postures in orientations that are farther apart, this fits with findings in the object recognition literature. It has indeed been shown that object identification becomes more orientation specific as objects in the stimulus set become more similar (e.g., Edelman, 1995, 1999; Murray, 1998; Newell, 1998; Humphreys and Forde, 2001; Rosselli et al., 2015). Human postures constitute a relatively homogeneous stimulus set, but the visual similarity becomes even larger when the postures are presented in the same or minimally different orientations. The trunk is the central part of the human body and the orientation of the limbs is specified vis-à-vis the trunk. Because the difference between the presented postures mostly is determined by the orientation of the limbs, more than by the orientation of the trunk, the visual projections of the trunk in different stimuli in the same orientations are very similar. Although the orientation of the trunk in some of the stimuli varies in the midsagittal plane (e.g., in a jumping down posture), the orientation in the midtransversal plane mostly remains constant (Figure 7).

**FIGURE 8:**
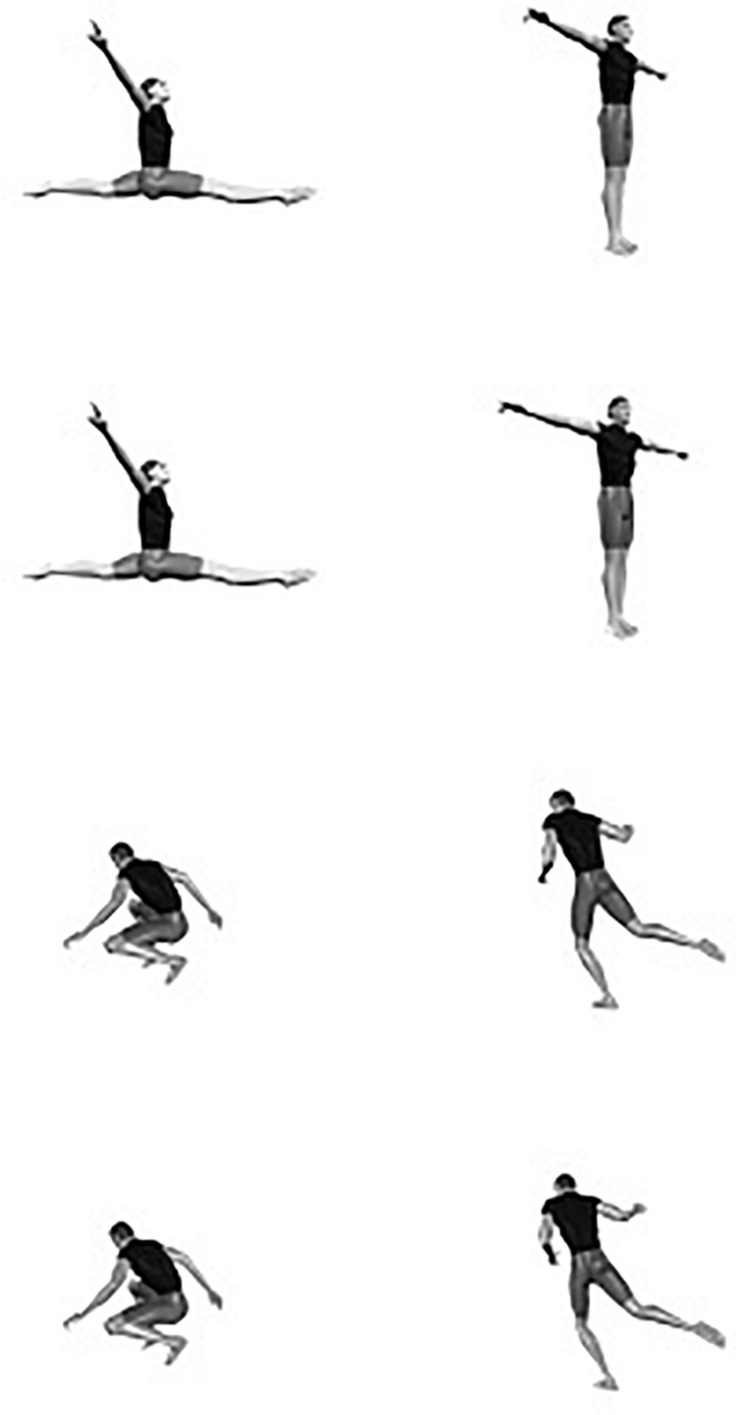
Illustration of the fact that small rotations result in less drastic changes in the projection of the trunk than large rotations (first row: two postures in more or less frontal view (75 and 60°) that differ by 15° in depth; second row: two postures in more or less frontal view (75 and 45°) that differ by 30° in depth; third row: two postures in more or less back view (255 and 240°) that differ by 15° in depth; fourth row: two postures in more or less back view (255 and 225°) that differ by 30° in depth).

The orientation differences in Experiment 4 of Daems and Verfaillie (1999) and Experiments 1 and 2 of the present study were realized by rotating the trunk in the midtransversal plane. Small rotations result in less drastic changes in the projection of the trunk than large rotations and therefore lead to less pronounced differences in the projection of the trunk and consequently cause smaller image changes between different postures (Figure 8).

It has been suggested (e.g., Perrett et al., 1991; Logothetis et al., 1995) that discrimination between visually similar objects leads to finer orientation tuning in neurons in infero-temporal cortex (IT). In order to identify body postures in a stimulus set with small orientation differences, the visual system might also rely on more finely tuned representations that result in stronger orientation-dependent effects. Stimulus sets with larger orientation differences then would lead to the activation of more broadly tuned representations causing more generalization.

The purpose of Experiment 3 was to examine to what degree the extent of the orientation differences between different body postures in the priming phase could explain the divergent results of Experiment 4 of Daems and Verfaillie (1999) and Experiment 1 of the present study. Two groups of participants were tested in the same possible/impossible decision task as used in the previous experiments. In one group participants were presented with body postures in a 15, 45, and 75° or in a 195, 225, and 255° orientation during the priming phase (relatively large orientation differences). In the second group postures were shown in a 45, 60, and 75° orientation or in a 225, 240, and 255° orientation in the priming phase (relatively small orientation differences). Both groups only saw postures in 75 and 255° orientations during the test phase. This design allowed us to investigate the degree to which long-term priming generalizes across a 30° orientation difference depending on the range of orientations used in the experiment.

### Method

#### Participants

Two groups of 60 subjects participated to the experiment. All participants had normal or corrected-to-normal vision and were tested individually. Subjects either were first or second year students who participated for course credit or were phd students.

#### Stimuli

**FIGURE 9:**
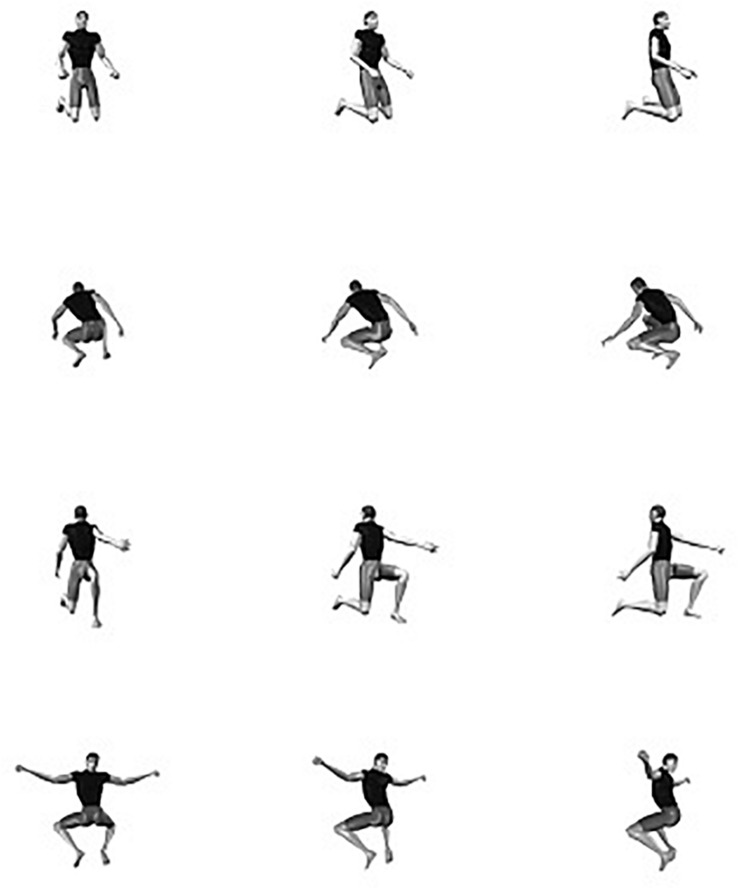
Examples of stimuli used in Experiment 3 (first row: possible posture in 15, 45, and 75° depth orientation; second row: possible posture in 195, 225, and 255° orientation; third row: impossible posture with lower body in 15, 45, and 75° orientation; fourth row: impossible posture with lower body in 195, 225, and 255°).

A total of 16 stimulus sets from Experiment 4 in Daems and Verfaillie (1999) were used. The first group of participants were presented with 12 sets. Three sets consisted of 24 anatomically possible postures that were oriented respectively 15,45, and 75° to the right (see Figure 9 for examples) and three sets consisted of 24 anatomically possible postures oriented 195, 225, and 255° to the right. The remaining six stimulus sets contained impossible postures with the lower body oriented 15, 45, and 75° oriented to the right in the first three sets and 195, 225, and 255° to the right in the other three sets. For the second group of participants, the 15 and 195° sets were replaced by the 60 and 240° sets of Experiment 4 in Daems and Verfaillie (1999). The other sets were the same as in the first group of participants. In addition to the experimental stimuli, there were 14 training stimuli.

#### Procedure

Participants were randomly assigned to the two groups. The first group participated in the condition with large orientation differences between the stimuli in the priming phase and the second group participated in the condition with small orientation differences in the priming phase. The stimuli in the test phase always were oriented 75 or 255° to the right. In the condition with large orientation differences stimuli in the priming phase either had the same orientation as in the test phase (75 or 255°), a 30° different orientation (45 or 225°), a 60° different orientation (15 or 195°), or there was no priming. In the condition with small orientation differences stimuli in the priming phase had the same orientation as in the test phase (75 or 255°), a 15 difference (60 or 240°), a 30° difference orientation (45 or 225°), or there was no priming. The smallest orientation difference between different stimuli in the priming phase therefore was 30° in the condition with large orientation differences and 15° in the condition with small orientation differences. In both conditions 12 anatomically possible and 12 impossible stimulus sets were divided over 4 groups of 6 stimuli that were rotated across long-term priming conditions and participants. Each participant was presented with 12 anatomically possible and 12 impossible postures in each long-term priming condition. Half of these 12 anatomically possible and 12 impossible postures were shown in a (more or less) frontal view (15, 45, and 75 or 45, 60, and 75°) and the other postures in a (more or less) back view (195, 225, and 255 or 225, 240, and 255°). The experiment always started with the training stimuli and 72 priming stimuli presented in a random order. After a short break, the 96 test stimuli were administered, also in a random order.

### Results

Reaction times below 200 ms or above 1400 ms (about 1% of the data), reaction times to stimuli that were not identified correctly in the priming phase or the test phase, and reaction times to three impossible postures that were identified correctly by less than half of the participants in both phases were removed from the data set. Mean RTs to anatomically possible and impossible postures can be found in Table 3. The MS errors in the subsequent ANOVAs give an impression of the variability in the data.

**TABLE 3:** Mean identification time (in ms) of possible and impossible human postures in the test phase of Experiment 3 as a function of context condition (large vs. small orientation differences) and long-term priming condition (same orientation, 15, 30, or 60° difference, or no priming).

	Possible postures		Impossible postures	
Priming condition	Large orientation difference	Small orientation difference	Large orientation difference	Small orientation difference
Same ori	640	628	709	728
15° difference	–	622	–	714
30° difference	642	644	710	718
60° difference	660	–	720	–
No priming	662	650	721	743

In a first series of analyses, RTs to stimuli in the 60° difference condition in the first group of participants (who were presented with relatively large orientation differences, but not the 15° difference) and RTs to stimuli in the 15° difference condition in the second group (who were presented with relatively small orientation differences, but not the 60° difference) were not taken into account. All other RTs from the test phase were entered in a participant and stimulus repeated-measures ANOVA with range of orientations (relatively large or small orientation differences in the priming phase) as between subjects variable or within stimuli variable, long-term priming condition (same orientation condition, 30° difference condition, or no priming) as within subjects variable or within stimuli variable, stimulus type (anatomically possible or impossible postures) as within subjects variable or between stimuli variable, and subject group or stimulus group as between subjects variable or between stimuli variable.

In the subject analysis as well as in the stimulus analysis, there was a main effect of stimulus type, *F*_1_(1,112) = 151.84, *MSE* = 7070, *p* < 0.001; *F*_2_(1,85) = 44.80, *MSE* = 17608, *p* < 0.001, a main effect of long-term priming condition, *F*_1_(2,224) = 11.36, *MSE* = 1945, *p* < 0.001; *F*_2_(2,170) = 10.37, *MSE* = 1646, *p* < 0.001, and no main effect of range of orientations condition, *F*_1_(1,112) = 0.09, *MSE* = 44447, *p* > 0.70; *F*_2_(1,85) = 0.46, *MSE* = 1750, *p* > 0.50. The interaction between stimulus type and range of orientations was marginally significant in the subject analysis, *F*_1_(1,112) = 3.62, *MSE* = 7070, *p* < 0.06, and significant in the stimulus analysis, *F*_2_(1,85) = 9.75, *MSE* = 1750, *p* < 0.01. Participants identified anatomically possible postures faster and impossible postures slower in the condition with small orientation changes than in the condition with large orientation changes. There was no significant interaction between stimulus type and long-term priming condition, *F*_1_(2,224) = 1.17, *MSE* = 1935, *p* > 0.30; *F*_2_(2,170) = 1.53, *MSE* = 1645, *p* > 0.20, between long-term priming condition and range of orientations, *F*_1_(2,224) = 0.03, *MSE* = 1945, *p* > 0.90; *F*_2_(2,170) = 0.00, *MSE* = 1668, *p* > 0.90, and between stimulus type, range of orientations, and long-term priming condition, *F*_1_(2,224) = 1.96, *MSE* = 1935, *p* > 0.10; *F*_2_(2,170) = 1.63, *MSE* = 1668, *p* > 0.10.

Even though stimulus type only interacted with range of orientations condition and not with long-term priming, separate analyses were performed for the anatomically possible and impossible postures. Differences between the same orientation condition, the 30° difference condition, and the condition without priming were evaluated by means of Dunn’s multiple comparison procedure. In the possible posture condition with large orientation differences, the difference between RTs in the same orientation condition and the condition without priming and in the condition with 30° different orientations and the condition without priming were significant in the subject and the stimulus analysis, *tD*_1_ = 2.53, *MSE* = 2187, *p* < 0.05; *tD*_2_ = 2.83, *MSE* = 1783, *p* < 0.05, and *tD*_1_ = 2.66, *MSE* = 1777, *p* < 0.05; *tD*_2_ = 3.47, *MSE* = 817, *p* < 0.01. In the condition with small orientation differences, we observed a quasi opposite effect. RTs in the same orientation condition differed significantly from RTs in the no-prime condition both in the subject and the stimulus analysis, *tD*_1_ = 3.73, *MSE* = 986, *p* < 0.01; *tD*_2_ = 2.63, *MSE* = 1543, *p* < 0.05, but the RT difference between the 30° different condition and the no-prime condition was not significant in both analyses. The difference between the same orientation condition and the 30° different condition was significant in the subject analysis, *tD*_1_ = 2.89, *MSE* = 870, *p* < 0.05, but not in the stimulus analysis.

The data indicate that long-term priming effects are modulated by the magnitude of the orientation differences between the stimuli in the priming phase. This is supported when also reaction times to stimuli in the 60° difference condition in the group of participants who were presented with large orientation differences and the reaction times to stimuli in the 15° difference condition in the group of participants who were presented with small orientation differences were taken into account (Table 3). The condition with 30° orientation differences resulted in significant long-term priming when the smallest orientation difference in the priming phase was 30°, but not when the smallest difference was 15°. The 60° different condition in the condition with large orientation differences did not result in long-term priming and the 15° different condition in the condition with small orientation differences resulted in a strong long-term priming effect, *tD*_1_ = 4.03, *MSE* = 1455, *p* < 0.01; *tD*_2_ = 3.27, *MSE* = 1472, *p* < 0.01. In the condition with large orientation differences as well as in the condition with small orientation differences a linear trend was observed (*F*_1_(1,56) = 10.79, *MSE* = 1929, *p* < 0.01; for the condition with large orientation difference, and *F*_2_(1,44) = 11.59, *MSE* = 1728, *p* < 0.01, for the condition with small orientation differences). Other trends were not significant.

In the condition with impossible postures (Table 3), no reliable differences or significant trends were found in the condition with large orientation differences. In the condition with small orientation differences there was a difference between the no-priming condition and the 15° difference condition in the subject and the stimulus analysis, *tD*_1_ = 4.55, *MSE* = 1272, *p* < 0.01; *tD*_2_ = 2.68, *MSE* = 1948, *p* < 0.05, and between the no-prime condition and the 30° difference condition in the stimulus analysis, *tD*_2_ = 2.84, *MSE* = 1712, *p* < 0.05.

A subject and stimulus analysis on the RTs in the priming phase (Table 4) indicated that the long-term priming effects in the test phase probably were not caused by differences in initial identification times. The RTs below 200 ms or above 1700 ms (about 1% of the data), the RTs of incorrect answers, and the RTs to 3 impossible postures that were recognized by less than half of the participants were discarded from the analysis. In both the subject and the stimulus analysis, there was a main effect of stimulus type, *F*_1_(1,112) = 25.09, *MSE* = 11720, *p* < 0.001; *F*_2_(1,85) = 12.82, *MSE* = 20676, *p* < 0.001, and of long-term priming condition, *F*_1_(1,112) = 6.30, *MSE* = 5334, *p* < 0.05; *F*_2_(1,85) = 6.23, *MSE* = 5192, *p* < 0.05, and no main effect of range of orientations condition. Stimulus type significantly interacted with range of orientations condition, *F*_1_(1,112) = 7.09, *MSE* = 11720, *p* < 0.01; *F*_2_(1,85) = 23.71, *MSE* = 3266 *p* < 0.001. There were no other significant two-way or three-way interactions.

**TABLE 4:** Mean identification time (in ms) of possible and impossible human postures in the priming phase of Experiment 3 as a function of context condition (large vs. small orientation differences) and long-term priming condition (same orientation, 15°, 30°, or 60° difference).

	Possible postures		Impossible postures	
Priming condition	Large orientation difference	Small orientation difference	Large orientation difference	Small orientation difference
Same orientation	919	889	944	968
15° difference	–	912	–	956
30° difference	929	918	950	991
60° difference	939	–	1019	–

In an analysis of the mean identification times to anatomically possible postures in the priming phase as a function of long-term priming condition and range of orientations condition there was only one reliable difference in the subject analysis. Participants were faster to respond in the same-orientation condition (75 and 255° orientations) than in the 30° difference orientation condition (25° and 225° orientations) in the condition with small orientation differences, *tD*_1_ = 2.68, *MSE* = 3461, *p* < 0.05. This implies that on average 45 and 225° orientations in this condition were viewed for a longer period of time than the 75 and 255° orientations. Yet, these conditions resulted in less pronounced long-term priming effects than in the condition with large orientation differences. It is therefore improbable that this difference between the same-orientation and the 30° difference condition in the priming phase was responsible for the range-of-orientations-dependent long-term priming effects observed in the test phase.

The analysis of the mean identification times for the impossible postures in the priming phase as a function of range of orientations condition and long-term priming condition indicated that in the condition with large orientation differences participants reacted more slowly in the 60° difference condition (15 and 195° orientation) than in the two other conditions (*tD*_1_ = 4.53, *MSE* = 8096, *p* < 0.01; *tD*_2_ = 5.18, *MSE* = 6276, *p* < 0.01 for the comparison with the same-orientation condition and *tD*_1_ = 4.10, *MSE* = 8409, *p* < 0.01; *tD*_2_ = 5.49, *MSE* = 5402, *p* < 0.01 for the comparison with the 30° difference condition). In the condition with small orientation differences impossible postures were identified faster in the 15° orientation difference condition (60 and 240° orientations) than in the 30° orientation difference condition (45° and 225° orientations), *tD*_1_ = 2.57, *MSE* = 5390, *p* < 0.05; *tD*_2_ = 2.76, *MSE* = 5364, *p* < 0.05. However, in the condition with large orientation differences as well as in the condition with small orientation differences there were no indications that RTs in the test phase were influenced by these initial identification differences.

We performed additional analyses on RTs for anatomically possible poses and for impossible poses in the priming phase with long-term priming condition, range of orientations condition, and global orientation (frontal or back view) as independent variables, but there were no significant interaction effects.

### Discussion

Experiment 3 showed that long-term priming is influenced by the extent of the orientation differences between the stimuli in the priming phase. When the orientation difference between stimuli was at least 30°, there were significant long-term priming effects in the 30° different-orientation condition. When a number of stimuli only differed by 15°, no long-term priming was observed in the same 30° different-orientation condition. These results can be interpreted in at least two ways. First, it is possible that the presence of a large 60° orientation difference resulted in broader tuning of the representational system, leading to more generalization over orientations. However, this explanation is improbable. The presence of a 60° different orientation condition in Experiment 4 of Daems and Verfaillie (1999) did not result in generalization over a 30° orientation difference. Moreover, participants in the condition with small orientation differences were also confronted with large orientation differences between different postures by the use of frontal and back views.

Apparently, not the presence of a 60° difference in the condition with large orientation differences, but the presence of a 15° difference in the condition with small orientation differences was crucial. Small orientation differences in the priming phase of a long-term priming experiment seem to result in finer orientation tuning of the representations that are used to identify body postures. This is remarkable. As can be observed in Figure 8, a 15° orientation difference is very subtle. Moreover, participants saw different postures in different orientations. Also, it was not necessary to attend the global orientation of the postures to be able to perform the possible/impossible decision task. Nevertheless, the visual system takes into account the size of the orientation differences between postures. In the case of relatively large differences more broadly tuned representations come into play leading to less specific long-term priming, while in the case of small differences finer tuning occurs leading to relatively stronger orientation-dependent priming.

It has been shown before that the extent of generalization for a stimulus in a particular orientation is variable and that it depends on the circumstances under which identification takes place. For example, when two orientations are connected by apparent motion, representations are tuned in such a way that short-term priming between the two orientations is facilitated, whereas priming outside the movement path is inhibited (Kourtzi and Shiffrar, 1997, 1999; see Cutting and Kozlowski, 1977; Loula et al., 2005; Prasad and Shiffrar, 2009, for examples of related research on the identification of people on the basis of their movement). Motion therefore influences the size and the nature of the generalization field around visual stimuli. The results of Experiment 3 indicate that the same holds for stimulus context (the range of orientations). Generalization fields shrink as the orientation difference between the to be identified stimuli decreases.

This observation fits with findings on object perception. Indeed, it has been reported repeatedly that object recognition becomes more orientation specific as the similarity of the objects in the stimulus set is more pronounced (Edelman, 1995; Murray, 1998; Newell, 1998, also see Perrett et al., 1991; Logothetis et al., 1995). Edelman (1999) explains this effect in a model in which similarity is represented in terms of distances. Similar orientations of an object are close to each other in an orientation space and the orientation spaces of similar objects are close to each other in a shape space. Therefore, the representation of an object in a particular orientation is codetermined by the representation of similar objects in the same orientation (also see Gauthier and Tarr, 1997; Tarr and Gauthier, 1998), making discrimination between similar objects in similar orientations more difficult.

**FIGURE 10:**
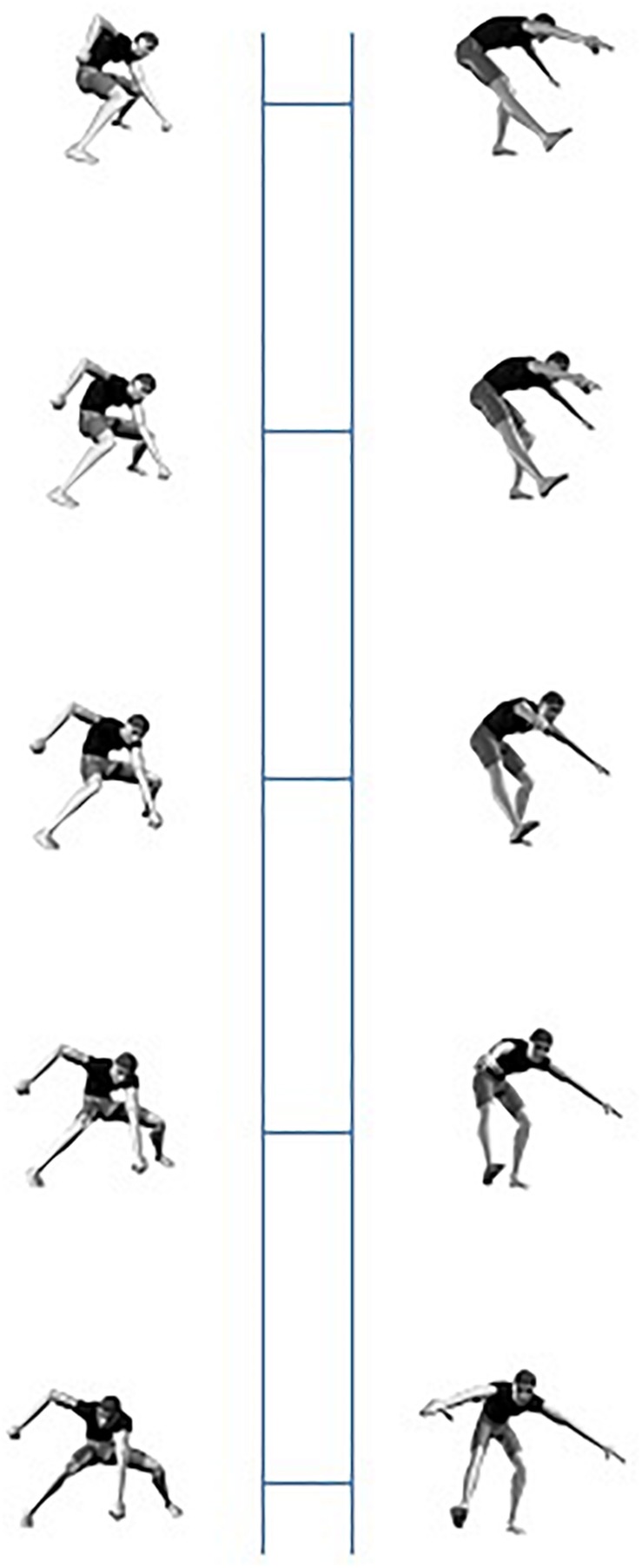
Common representational space of two human body postures (each column depicts a particular postures in four different depth orientations; in each row two similar but distinct postures in the same depth orientation).

In Figure 10 a similar model for the perception of body postures is depicted. In this model the similarity between different orientations of two different postures is shown. This representation shows that (the projection of) different postures in the same or minimally different orientations are visually more similar than different postures (or even the same posture) in strongly different orientations.

The model in Figure 10 should not be conceived of as a static memory model for specific body postures. A considerable number of participants in Experiment 3 probably never encountered the specific postures as shown in Figure 10. Apart from a few exceptions (e.g., walking, crawling), for most body postures participants cannot access a specific memory model. Instead, the visual system utilizes a general dynamic body scheme that can be set into the correct orientation and posture. In the case of close orientations of similar postures, this process probably proceeds in a more similar manner, as a result of which the dynamic representations involved are overlapping to a considerable degree. In this sense, the distances in the model in Figure 10 stand for the relative similarity between pairs of body postures in different orientations on the one hand, and the relative overlap of the dynamic representations on the other hand.

The context (i.e., the range of orientations used) dependent long-term priming effects in Experiment 3 suggest that the underlying representational system is flexible and that posture identification is optimized by adapting the degree of overlap between the representations to the stimulus context (or more generally, the task at hand). A stimulus set with large orientation differences between different stimuli gives rise to more broadly tuned representations than a stimulus set with small orientation differences. Apparently, representations are more finely tuned when identification involves making finer discriminations. Representations that are smaller tuned are closer to the original stimulus and cause less generalization. By using more narrowly tuned representations, it is avoided that similar stimuli in similar orientations lead to the activation of strongly overlapping representations that make precise identification difficult.

Tuning of representations or generalization fields can be conceptualized as an adaptation (during the task) of the manner in which neurons and neuron populations are activated. When, as the task proceeds, it becomes obvious that the normal activation spreading of neuronal populations results in a larger overlap for different stimuli, a stronger criterion is set, as a result of which smaller tuning is established. In a long-term-priming experiment this adaptation primarily occurs during the priming phase. When confronted with a stimulus in the priming phase groups of neurons are tuned as a function of a number of stimulus characteristics. When during the test phase the same stimulus with the same characteristics is shown again, the identification threshold is reached faster. If, however, there is a significant change in a stimulus characteristic, partly different neurons are activated, resulting in no or less facilitation. The stimulus context (e.g., range of orientations used) probably codetermines the breadth of tuning of the neurons or the extent of activation spreading over neurons and neuron populations.

## General Discussion

How do observers identify human body postures? Is it the case that for all possible orientations of a body postures a single orientation-independent, object-centered memory model can be accessed in order to identify the posture (e.g., Marr and Vaina, 1982)? Or is identification accomplished via different orientation-specific representations. The results of Daems and Verfaillie (1999) and the present study support the latter hypothesis^1^.

We were not able to directly address the inconsistency between the present Experiment 1 (i.e., priming across a 30° difference between and test posture) and research previously described by Daems and Verfaillie, 1999 (priming across a 15° difference), but through our quest to explain the difference, in Experiments 2 and 3 we were able to elucidate the flexibility of the posture representation system in the brain (e.g., relative independence of actor identity).

As already indicated, the findings in the long-term priming experiments are in line with observations on object recognition. On the one hand, this suggests that the visual system uses similar mechanisms and representations to identify objects and postures in different orientations. On the other hand, identification of body postures seems to be special. When observers are confronted with a human figure, it is probable that a general body scheme is activated that is adjusted in the correct posture and orientation. In the experiments reported in Daems and Verfaillie (1999) and Experiment 3 of the present study (in Experiments 1 and 2 the impossible postures only served as filler stimuli and were not systematically manipulated) there was no long-term priming for impossible postures. This suggests that the dynamic representations that are used for the identification of human postures are orientation specific and constrained by the biomechanical limits of the human body (also see Kourtzi and Shiffrar, 1999; Candidi et al., 2008; Cross et al., 2010; Schouten et al., 2011; Davila et al., 2014; Heenan and Troje, 2014).

Neurophysiological studies confirm the existence of a body-specific representational system (e.g., Downing et al., 2001; Peelen and Downing, 2007; Hodzic et al., 2009; Van der Wyk et al., 2009). Indeed, static pictures of human bodies activate other brain regions than images of objects. For instance, in a PET study of Peigneux et al. (2000) presentation of objects primarily resulted in activation in the occipital and fusiform gyrus, whereas body postures activated parts of the lateral occipitotemporal junction and area MT/V5.

There is evidence that region MT/V5 is strongly associated with motion perception (e.g., Howard et al., 1996), but also that it is involved in the perception of static pictures that imply motion (e.g., Peuskens et al., 2005; Urgesi et al., 2006; Pavan et al., 2011). Activation of this part of the visual cortex by the presentation of static postures probably reflects the importance of movement (even if only implied in the perception of human body postures; but see Lorteije et al., 2011). Body postures mostly are part of a movement or action sequence in which the exact position and orientation of the body as a whole and of the different body parts with respect to each other change. Integration of different postures and action phases therefore form another important component of action perception. An experiment reported by Verfaillie and Daems (2002) indeed showed that action representations are more broadly tuned in the direction of motion. On each trial in the priming phase, participants were presented with pairs of brief action animations (performed by two different human models) and had to decide whether the actions were the same or not. In the test phase subjects saw static possible or impossible postures (as in Daems and Verfaillie, 1999, and the present study). Reliable priming was observed for test postures that were preceded by a priming animation in which the figure would have reached the test posture if the priming animation would have lasted longer, but not for test postures preceded by a priming animation in which the figure would have been if the priming animation had started earlier (in comparison to a condition in which the test posture was not seen in a related priming animation). This observation indicates that movement is important to achieve generalization and anticipation to future action phases.

Some action sequences consist of changes in the global orientation of the human figure who performs the action (e.g., rotating movement as in a pirouette). In this case integration of different action phases boils down to integration of different orientations. In short-term priming experiments, it has been shown before (e.g., Kourtzi and Shiffrar, 1997, 1999; see Manera et al., 2013, for related research) that movement facilitates generalization to and anticipation of new orientations. Since human observers in daily life mostly are confronted with subsequent orientations of body postures as a result of their own movement or the movement of the observed figure, this mechanism allows identification that makes abstraction of orientation on the basis of orientation-specific representations.

In sum, the dynamic orientation-specific representations supporting posture perception are flexible and dependent on stimulus and task context. This allows the visual system to achieve a broad range of tasks. Successful identification of highly similar body postures in similar orientations probably is best supported by more finely tuned representations, whereas anticipation of future orientations and action phases (e.g., Verfaillie and Daems, 2002; Manera et al., 2013) and other tasks that are based on generalization would be more efficient with broader orientation tuning.

## Data Availability Statement

The datasets generated for this study are available on request to the corresponding author.

## Ethics Statement

The studies involving human participants were reviewed and approved by the Ethics Committee of the Faculty of Psychology and Educational Sciences. Written informed consent for participation was not required for this study in accordance with the national legislation and the institutional requirements.

## Author Contributions

Both authors contributed to framing the study theoretically, designing and executing the experiments, analyzing the data, interpreting the results, and drawing conclusion.

## Conflict of Interest

The authors declare that the research was conducted in the absence of any commercial or financial relationships that could be construed as a potential conflict of interest.

## Footnotes

1 Note that the link between low and high-level processing on the one hand and early and later processing mode is a hypothesis and not every researcher agrees with the link (see Sekunova et al., 2013; Brooks et al., 2016, 2018; for relevant literature in the domain of body adaptation).
